# Bioactive Compounds in Fruits and Vegetables: From Matrix to Metabolism

**DOI:** 10.3390/foods15193546

**Published:** 2026-10-04

**Authors:** Jian Li, Junjie Li

**Affiliations:** College of Ocean Food and Biological Engineerin, Jimei University, Xiamen 361021, China; 202514832007@jmu.edu.cn

The notion that plant-rich diets confer long-term health advantages is no longer a hypothesis but a well-established principle in nutritional science. Fruits and vegetables, in particular, are universally endorsed as cornerstones of healthy eating patterns, not merely for their macronutrient and vitamin profiles, but for the vast array of secondary metabolites they contain—collectively referred to as bioactive compounds. These include polyphenols, carotenoids, glucosinolates, and flavonoids, among others, and their potential to mitigate chronic conditions such as cardiovascular disease, type 2 diabetes, and certain cancers has drawn sustained research interest over the past decades [1].

Yet, for all the enthusiasm surrounding these natural molecules, the pathway from the plant cell to a measurable physiological effect in humans is far from straightforward. A range of hurdles must be overcome: the efficiency of extraction, the stability of compounds during processing, their bioaccessibility in the gastrointestinal tract, and ultimately their absorption, distribution, and metabolism. It is at this intersection—where food science meets human physiology—that the greatest gaps in our knowledge remain. This Special Issue, “Bioactive Compounds in Fruits and Vegetables: From Matrix to Metabolism,” seeks to bring together studies that confront these challenges from multiple angles, offering both methodological advances and mechanistic insights.

The initial step of isolating bioactive compounds from plant matrices is a critical determinant of subsequent research and application. Conventional solvent-based methods, while widely employed, are increasingly scrutinized for their environmental footprint and their potential to degrade heat- or oxygen-labile constituents. In response, a growing body of work has turned to greener and more efficient alternatives. Ultrasound- and microwave-assisted extractions have gained traction, as have enzyme-assisted approaches that operate under milder conditions [2]. But beyond the technology itself, the choice of plant material matters: genetic variation, harvest time, and growing conditions all shape the phytochemical landscape, as several studies in this Special Issue illustrate.

Once extracted, the biological utility of these compounds is heavily constrained by their bioavailability—or more precisely, their lack thereof. Poor aqueous solubility, limited membrane permeability, and extensive first-pass metabolism mean that many dietary bioactives reach systemic circulation in only trace amounts. This has prompted a surge of interest in strategies to enhance their delivery. Processing technologies such as high-pressure homogenization and microfluidization have been shown to modify food matrices in ways that improve the release of phenolic compounds during digestion. Meanwhile, formulation approaches—including encapsulation, emulsion systems, and co-delivery with absorption enhancers—offer complementary routes to improving the fraction that ultimately reaches target tissues [3].

Equally important is the question of how these compounds act once they enter the body. The antioxidant and anti-inflammatory properties of fruit- and vegetable-derived bioactives are well recognized, but recent work has moved beyond simple radical-scavenging assays to dissect their influence on specific signaling cascades. For instance, certain polyphenols have been shown to modulate the IRS-1/PI3K/Akt pathway, improving insulin sensitivity in peripheral tissues, while others interfere with lipid digestion by directly inhibiting pancreatic lipase [4]. These mechanistic studies are essential not only for validating health claims but also for identifying the most promising candidates for functional food development.

The translation of this knowledge into consumer-relevant products is perhaps the most tangible goal of the field. Whether in the form of fortified beverages, dietary supplements, or novel plant-based formulations, the incorporation of bioactive compounds into functional foods requires careful attention to sensory properties, stability during shelf life, and regulatory compliance [5]. The contributions to this Special Issue highlight both the opportunities and the trade-offs inherent in this process, and they underscore the value of interdisciplinary collaboration between food technologists, nutritionists, and biochemists.

The contributions to this Special Issue collectively illustrate how research on food bioactives is moving from compositional characterization toward a more integrated understanding of biological function, bioavailability, processing, and practical application. Several studies focused on the physiological and molecular activities of food-derived components. Polypeptides from *Volvariella volvacea* were shown to alleviate alcohol-induced liver injury, with multi-omics analyses implicating the regulation of endoplasmic-reticulum stress and lipid and amino-acid metabolism [6], while *Praecitrullus fistulosus* extract improved glucose and lipid homeostasis in diabetic rats in association with enhanced insulin signaling, including GLUT-4 and IRS-1 [7]. A more target-oriented approach was provided by the identification of pancreatic lipase-inhibitory peptides from *Chlorella pyrenoidosa*, among which VWTPI showed particularly strong inhibitory activity [8]. Importantly, biological potential also depends on whether active compounds become accessible at their sites of action, as demonstrated by the markedly compound-dependent intestinal transport of red grape skin polyphenols in a Caco-2 model [9]. Other contributions emphasized the importance of raw-material characteristics and processing in determining functional quality. The nutritional and phytochemical profiles of glassworts varied substantially with genotype and growing site [10], whereas enzymatic deastringency of sea buckthorn improved sensory characteristics through coordinated metabolic changes while retaining antioxidant activity [11]. These findings were further extended toward product development: stable non-fermented plant-based probiotic beverages were obtained while maintaining probiotic viability and acceptable physicochemical and sensory properties [12], and targeted LC-MS/MS profiling was used to exploit ingredient complementarity in designing a plant-based smoothie with a diversified polyphenolic profile [13].

Together, these studies show that the functional value of food bioactives is determined not by composition alone, but by the interplay among source characteristics, processing, bioaccessibility, molecular activity, and formulation. Collectively, they provide a broader perspective on how bioactive-rich foods can be translated into practical food applications. We hope this collection will serve as a useful reference for researchers and practitioners alike, and that it will stimulate further work aimed at bridging the divide between the bioactive potential of plant foods and their real-world impact on human health.

## Data Availability

No new data were created or analyzed in this study. Data sharing is not applicable to this article.
